# Case Report: A case of Vulvar Paget’s disease and literature review

**DOI:** 10.3389/fonc.2025.1648891

**Published:** 2025-10-31

**Authors:** Guojie Wang

**Affiliations:** The Department of Obstetrics and Gynecology, West China Second University Hospital of Sichuan University, Chengdu, China

**Keywords:** Vulvar Paget’s disease, pathologic characteristic, diagnosis, treatment, prognosis

## Abstract

**Introduction:**

Vulvar Paget’s disease (VPD) is a rare vulvar malignant tumor commonly observed in postmenopausal females. VPD cases are mainly treated by surgery, and the postoperative recurrence rate is high. Owing to the absence of any specific clinical manifestations, VPD is often misdiagnosed as eczematous skin lesions, which leads to diagnostic delay.

**Case presentation:**

A 63-year-old woman experienced vulvar itching for over 4 months after 15 years of menopause. Computed tomography scans revealed a slightly friable vulva. Gynecological examination detected that the bilateral labia majora, especially the right labia majora, were exposed to light skin pigmentation; a red spot with a diameter of 5cm was observed outside the right labia majora, with visible scratch marks. The pathological results of the vulvar biopsy indicated Paget’s disease. The patient received local vulvectomy, vulvar skin flap transplantation, and vulvar plastic surgery. Routine gynecological examination and radiological examination indicated negative results 3 years after the surgery. Moreover, neither local recurrence nor distant metastasis was recorded.

**Conclusion:**

VPD is commonly misdiagnosed as its clinical manifestations are nonspecific, mimicking other dermatological diseases. The diagnosis of VPD relies on pathological examination. Surgical treatment is preferred for its treatment, but the recurrence rate is high. Hence, early diagnosis and postoperative follow-up are critical in VPD treatment. Early diagnosis and treatment can improve the survival and quality of life of VPD patients.

## 1 Introduction

Extramammary Paget’s disease (EMPD) is a rare intraepithelial adenocarcinoma, accounting for approximately 6.5% of all Paget’s disease cases (1–4). EMPD predominantly occurs in areas with abundant apocrine glands, with the vulva being the most common site, followed by the perianal region, scrotum, penis, and axilla (5–7). Vulvar Paget’s disease (VPD), which accounts for over 50% of EMPD cases (8), primarily affects postmenopausal women (9, 10). Clinically, VPD presents as red vulvar plaques with well-demarcated borders, often accompanied by pruritus, localized pain, or a burning sensation (11). Due to its resemblance to eczematous lesions, it is frequently misdiagnosed (12). Approximately 15% of patients are asymptomatic (13). A vulvar skin biopsy is the diagnostic gold standard. Surgical excision remains the primary treatment. In most cases, the disease remains confined to the epithelium, resulting in a favorable prognosis, with a five-year survival rate exceeding 90% (14). However, dermal invasion significantly worsens the prognosis, as it increases the risk of malignancy and distant metastasis (15). The recurrence rate of VPD is relatively high after surgery, so long-term follow-up is crucial. Here, we report our experience in treating a patient with VPD who underwent surgery and did not experience recurrence or metastasis during a three-year follow-up.

## 2 Case report

**Figure 1:**
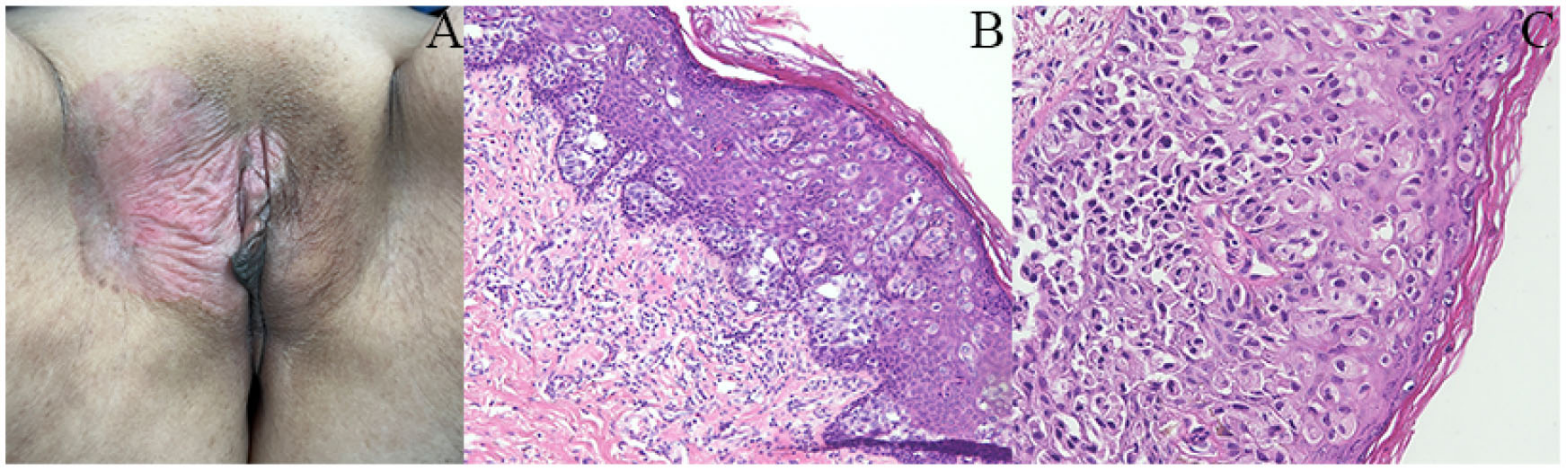
Clinical and histopathological features of the patient. **(A)** Gynecological examination revealed a 5-cm red lesion with visible scratch marks in the right-side labia majora. **(B, C)** Histopathological section indicated dysplastic stratified squamous epithelium showing basal acantholysis.

**Figure 2:**
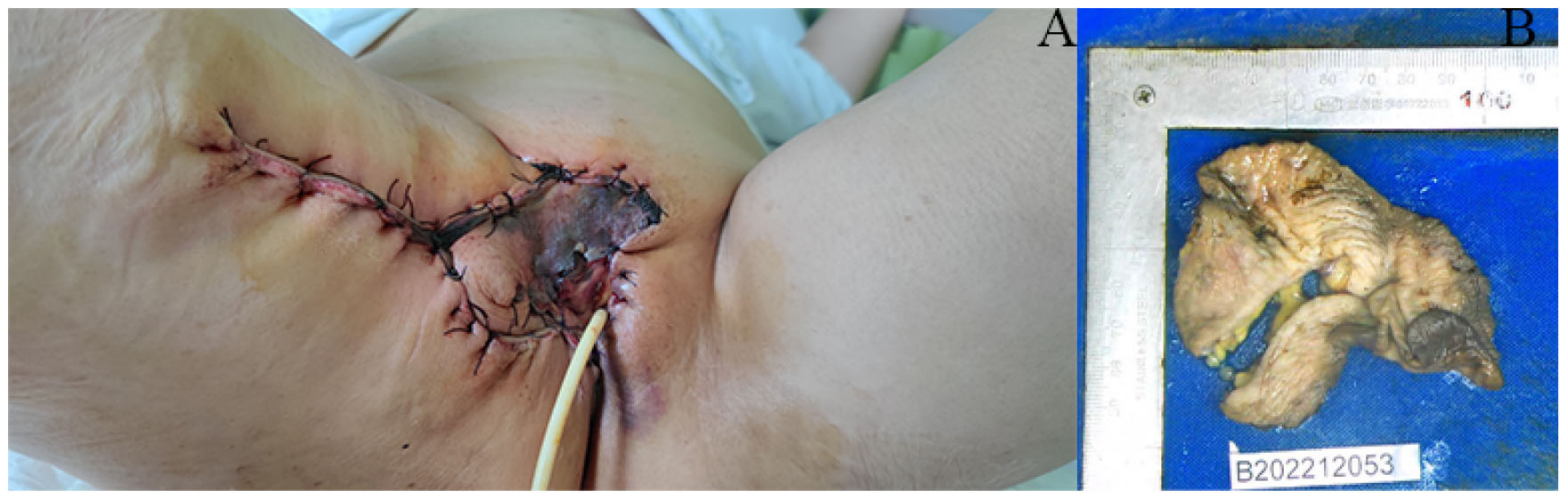
Surgery specimen of the patient. **(A, B)** Surgery was performed on the lesion, which involved local vulvectomy with skin flap transplantation and vulvar reconstruction.

**Figure 3:**
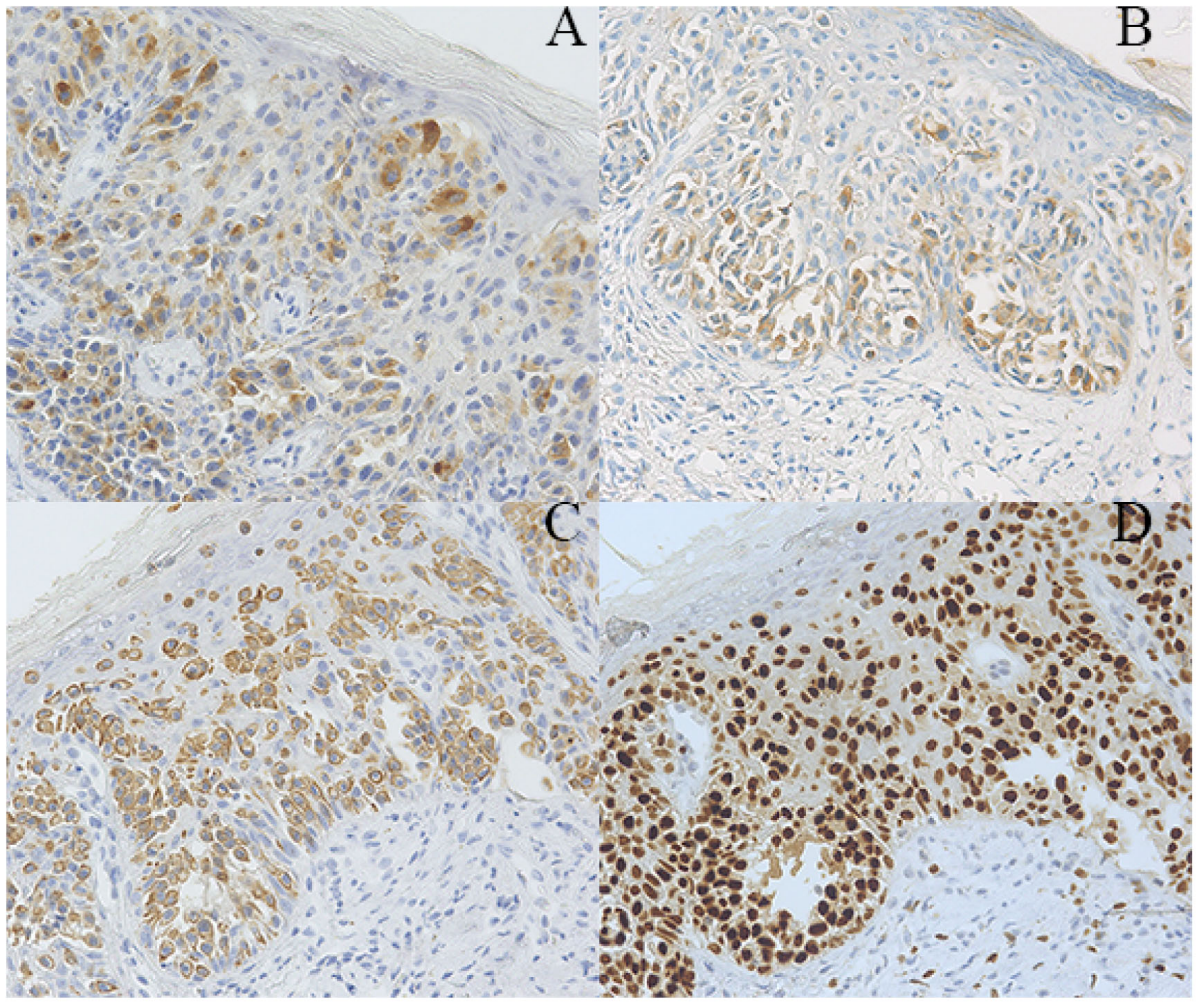
Pathological findings of the patient. **(A–D)** Immunohistochemical staining showing tumor cells positive for GCDFP-15 **(A)**, CEA **(B)**, CAM5.2 **(C)**, and GATA3 (magnification x200).

A 63-year-old postmenopausal woman presented with vulvar pruritus persisting for over four months. She had been menopausal for 15 years, with a history of hypertension for five years. She had not experienced childbirth. A computed tomography scan revealed mild roughness of the vulva. Gynecological examination showed light pigmentation of the bilateral labia majora, particularly on the right side, where a 5-cm red lesion with visible scratch marks was observed (**Figure 1A**). Histopathological section indicated dysplastic stratified squamous epithelium with basal acantholysis (**Figures 1B, C**). The patient underwent local vulvectomy with skin flap transplantation and vulvar reconstruction (**Figures 2A, B**). Postoperative pathology confirmed VPD with positive IHC markers for GCDFP-15, CEA, CAM5.2, and GATA3 (**Figures 3A–D**). Follow-up included regular gynecological and radiological assessments. After three years, there was no evidence of local recurrence or distant metastasis.

## 3 Discussion

VPD is the most common form of EMPD, accounting for 1-2% of all vulvar tumors (16). First described by Dubreuilh in 1901, VPD typically originates in the labia majora and can extend to the pubic region, inguinal folds, perineum, medial thighs, labia minora, and vagina. VPD is classified as either primary or secondary based on its histocytological origin (17). Primary VPD arises from basal epithelial cells, often involving adjacent skin appendages. In some cases, it originates from skin appendages or sweat glands, which may give rise to adenocarcinomas. Secondary VPD results from epidermal metastasis or direct invasion by adenocarcinomas of the anal, rectal, or urogenital epithelium. VPD predominantly affects postmenopausal women over the age of 60, and its non-specific symptoms often delay diagnosis, contributing to disease progression.

The exact etiology of VPD remains unclear, with potential associations with seborrheic dermatitis, superficial fungal infections, Bowen’s disease, and superficial basal cell carcinoma (18). Some studies suggest a link between VPD and EMPD, proposing that EMPD may originate from ectopic mammary glands in the vulva (19). HER2/neu overexpression or amplification is related to the pathogenesis of VPD (20). It has been demonstrated to be a basic pathogenic pathway of VPD. Garganese et al. reported that 21% of non-invasive VPD cases and up to 45.5% of invasive VPD cases were exposed to HER2/neu re-expression or amplification (21). Moreover, HER2/neu overexpression or amplification is associated with a poor prognosis, considering that it involves tumor invasion, lymph node metastasis, and recurrence (22–25). Ogawa et al. reported that 19.4% of metastatic VPD cases were exposed to HER2 overexpression, which was achieved via CISH (26).

Clinically, VPD typically presents as a well-defined red or brown plaque, a few centimeters in size, with a smooth surface. Patients often experience pruritus, pain, or a burning sensation (27). In advanced stages, lesions may ulcerate, bleed, or exhibit edema, crusting, and nodularity, with potential regional lymph node enlargement (28). Lesions can be solitary or multiple, and unilateral or bilateral. The VPD demonstrated non-specific clinical manifestations, leading to confusion with eczema, malignant melanoma, vulvar squamous intraepithelial lesions, vulvar squamous cell carcinoma, chronic simple lichen, and atrophic sclerosing lichen. As a result, corticosteroids and/or antifungal drugs are used, resulting in lesion deterioration. Therefore, biopsy is considered when the local drug efficacy is poor or the development of vulvar lesions is highly likely. The gold standard for diagnosing VPD is a pathological examination, including skin biopsy and cytologic analysis of vulvar lesion scrapings. Gross examination often reveals epidermal thickening with occasional fibroepitheliomatous changes (29). Microscopically, VPD is characterized by solid, nested, or glandular structures composed of large, oval, or polygonal Paget cells, which have abundant cytoplasm, transparent vacuoles, and prominent nucleoli (30). Paget cells are dispersed or clustered within the epithelium; they are typically located near the basal layer. After HE staining, the cytoplasm appears grey-blue. Special staining can facilitate the identification of Paget cells. For instance, the cytoplasm of Paget cells, which contains neutral and acidic mucus, is stained positively after periodic acid–Schiff (PAS) staining, which significantly helps distinguish VPD from other vulvar diseases (31, 32). Immunohistochemistry is crucial for the diagnosis of VPD, and VPD cells typically express GCDFP-15, CEA, CK7, and Ca15–3 instead of CK20 or HMB-45. Melanoma cells are highly similar to Paget’s cells, whereas melanoma cells display overexpression of Mel-A, HMB45, and S-100 instead of GCDFP-15, CEA, and CK7 after IHC staining (33). Hence, Ca15–3 can serve as a biomarker for the differentiation of VPD from Bowen’s disease (34). Moreover, both HPV typing test and liquid-based cytology of papillomavirus-induced high-grade vulvar intraepithelial neoplasia were positive, although no etiological studies have demonstrated the relationship of VPD with HPV infection (35).

The incidence of VPD is rare, and large-scale studies are lacking, resulting in no standardized treatment approach. Currently, surgery is the primary treatment option. However, due to the extensive infiltration of Paget cells into the epidermis, complete removal is challenging, leading to high recurrence rates (36, 37). Postoperative recurrence rates for radical vulvectomy, radical semi-vulvectomy, and wide excision are reported at 15%, 20%, and 43%, respectively (16). Murata et al. have recommended a surgical margin of 2cm to reduce recurrence (38, 39). If interstitial infiltration exceeds 1mm or there is concern for adenocarcinoma, the surgical depth should extend to the fascial layer, with unilateral or bilateral inguinal lymph node dissection based on lesion location (40, 41). Additionally, Mohs micrographic surgery has shown promise in reducing recurrence rates (42). Bae et al. analyzed 90 cases treated with Mohs surgery and reported a 12.2% recurrence rate and an 83.6% five-year tumor-free survival rate (43). Similarly, Bruce et al. found a three-year recurrence rate of 6.7% for Mohs surgery, compared to 34.1% for traditional excision (44). Achieving complete negative margins with Mohs surgery often requires excising more than 5cm beyond the visible lesion in 97% of cases (45). In cases with large lesions, reconstructive surgery, such as skin grafts, local flaps, or muscle flap grafts, is often necessary. The complication rate for skin grafts after vulvectomy is approximately 6.8%, including sexual dysfunction and mobility issues (46). In this case, pathological examination confirmed VPD with positive immunohistochemical markers for GCDFP-15 and CEA. The patient underwent local vulvectomy, vulvar skin flap transplantation, and vulvar reconstruction. Intraoperative frozen section biopsy ensured negative margins. After three years of follow-up, regular gynecological exams and radiological assessments showed no recurrence or distant metastasis. Therefore, for women with VPD, local excision with negative margins provides favorable outcomes, and intraoperative frozen section biopsy is recommended to confirm margin status.

Imiquimod, a topical immune response modulator, has emerged as an alternative therapy for treating VPD (47). As a Toll-like receptor 7 agonist, it activates T cells and Langerhans cells, enhancing the immune response. Imiquimod exhibits direct anti-tumor activity by inhibiting cancer cell proliferation through the induction of autophagy and apoptosis (48, 49). Compared to extensive lesion excision, imiquimod may offer a more effective cure for VPD (50). In 2003, Wang et al. (51) was the first to report complete remission in recurrent VPD following topical imiquimod treatment. In a retrospective study by Machida et al. involving 63 patients with VPD, 73% achieved complete remission with no disease progression (52). The cumulative complete remission rates at 2, 4, and 6 months were 9.8%, 31.1%, and 71.6%, respectively. Despite these promising results, larger studies with long-term follow-up are needed to confirm the efficacy of imiquimod in VPD treatment.

Non-surgical treatments, such as radiotherapy, chemotherapy, and immunotherapy, are viable options for advanced-stage patients, particularly those unable to tolerate surgery. Radiation therapy is often used as an adjuvant treatment for patients with positive surgical margins, postoperative recurrence, or those who cannot undergo surgery (53, 54). A systematic review by Tagliaferri et al. of 195 patients treated with radiation therapy reported a complete remission rate of 92.6% and a recurrence rate of 33% (55). Iacoponi also found that postoperative radiotherapy in advanced cases reduced metastasis risk and extended survival (56). Chemotherapeutic agents such as 5-fluorouracil and bleomycin are sometimes used in VPD (57–59), though their efficacy remains unclear (60–62), and chemotherapy is not recommended as a primary treatment (63, 64). For advanced patients with VPD with human epidermal growth factor receptor 2 (HER2) positivity and distant metastasis, immunotherapy with trastuzumab combined with paclitaxel may be considered. Kimura et al. reported that one HER2-positive VPD patient with pulmonary lymph node metastases showed no malignant progression for 36 months following immunotherapy (59).

Recently, novel therapies such as CO_2_ laser treatment and photodynamic therapy have been explored for VPD management. However, CO_2_ laser treatment has limited effectiveness as a primary therapy, with recurrence rates of 31–67% for superficial lesions (65, 66). Photodynamic therapy, while innovative, can cause severe pain at the treatment site, making it challenging for some patients to complete the regimen, and is less effective in areas that are difficult to expose to light (67, 68).

## 4 Conclusions

VPD, although rare, predominantly affects postmenopausal women and presents with atypical symptoms, often resembling eczematous skin lesions. This can lead to delayed diagnosis and disease progression. Clinicians should carefully consider the patient’s history and clinical signs when VPD is suspected and perform a biopsy promptly for accurate diagnosis. Based on the pathological findings, individualized treatment plans should be developed. Given the high postoperative recurrence rate, long-term follow-up is essential for monitoring disease recurrence and ensuring timely intervention. In case of suspected recurrence, invasion, or metastasis, thorough gynecological examinations (including vulvar, vaginal, and digital rectal examinations) and complete radiological examinations (such as computed tomography and magnetic resonance imaging) should be conducted during the follow-up period to evaluate the overall condition of the patients.
